# Diet Sustainability Analyses Can Be Improved With Updates to the Food Commodity Intake Database

**DOI:** 10.3389/fnut.2022.868485

**Published:** 2022-06-27

**Authors:** Zach Conrad, Ashley Cyril, Corina Kowalski, Erin Jackson, Brittany Hendrickx, Jessie Jie Lan, Acree McDowell, Meredith Salesses, David C. Love, Troy Wiipongwii, Fang Fang Zhang, Nicole Tichenor Blackstone

**Affiliations:** ^1^Department of Kinesiology, William & Mary, Williamsburg, VA, United States; ^2^Global Research Institute, William & Mary, Williamsburg, VA, United States; ^3^College of Arts and Sciences, William & Mary, Williamsburg, VA, United States; ^4^Division of Agriculture, Food, and Environment, Friedman School of Nutrition Science and Policy, Tufts University, Boston, MA, United States; ^5^Division of Nutritional Epidemiology and Data Science, Friedman School of Nutrition Science and Policy, Tufts University, Boston, MA, United States; ^6^Department of Environmental Health and Engineering, Johns Hopkins Bloomberg School of Public Health, Johns Hopkins University, Baltimore, MD, United States; ^7^Center for a Livable Future, Johns Hopkins Bloomberg School of Public Health, Johns Hopkins University, Baltimore, MD, United States

**Keywords:** FCID, NHANES, sustainability, data integration, dietary patterns

## Abstract

Diet sustainability analyses inform policymaking decisions and provide clinicians and consumers with evidence-based information to make dietary changes. In the United States, the Food Commodity Intake Database (FCID) provides a crosswalk for integrating nationally representative data on food intake from the National Health and Nutrition Examination Survey (NHANES) with data on sustainability outcomes from other publicly available databases. However, FCID has not been updated since 2010 and does not link with contemporary NHANES data, which limits further advancements in sustainability research. This study fills this research gap by establishing novel linkages between FCID and NHANES 2011–2018, comparing daily per capita food intake with and without these linkages, and making these data publicly available for use by other researchers. To update FCID, two investigators independently established novel data linkages, a third investigator resolved discrepancies, and a fourth investigator audited linkages for accuracy. Dietary data were acquired from nearly 45,000 adults from 2001 to 2018, and food intake was compared between updated vs. non-updated FCID versions. Total food intake from 2011 to 2018 was 5–23% higher using the updated FCID compared to the non-updated version, and intake was over 100% higher in some years for some food categories including poultry, eggs, legumes, starchy vegetables, and tropical oils (*P* < 0.001 for all comparisons). Further efforts may be needed to create new food composition data to reflect new products and reformulations that enter the food supply over time. This study removes a barrier to further diet sustainability analyses by establishing a data crosswalk between contemporary NHANES and other publicly available databases on agricultural resource use, environmental impacts, and consumer food expenditures.

## Introduction

Diet sustainability analyses have increased in number over the past decade (1) in response to growing global awareness that food system transformation is needed to address concerns about human health, environmental impacts, food affordability, and social justice (2, 3). Unlike food system sustainability analyses, which focus broadly on the conditions and decisions that occur throughout a food system (e.g., production, processing, transport, and consumption), diet sustainability analyses focus more narrowly on the sustainability impacts of consumer food choices. As a result, these findings inform consumer-oriented policy action including the development of sustainable dietary guidance, and are directly relevant to clinicians and consumers seeking evidence-based information on how to make impactful dietary changes (4).

For example, a growing number of countries have adopted sustainable dietary guidelines and several more have attempted it, including the United States (US) (5). Over one-third of US consumers report that considerations of environmental sustainability are an important driver of their food choices, and nearly one-third report that it has had much more or somewhat more of an impact on their food purchasing decisions over the previous 10 years (6). Willets-Smith et al. (7) demonstrated that targeted dietary shifts among individuals motivated by health and environmental concerns (16% of the total population) can reduce GHG emissions by up to 6.7%, further demonstrating the potential impact of consumer behavior changes. It bears noting that dietary sustainability cannot solely be achieved by shifts in motivated consumers' behavior; it will require multi-faceted, population-level interventions (i.e., regulation, subsidies, changes in public procurement) (8).

Although most diet sustainability analyses have been conducted using data collected from other countries, the number of studies conducted using US-based data has increased (1) as data integration methods have improved (9). For example, Canning et al. (10) combined dietary data from the National Health and Nutrition Examination Survey (NHANES) with an environmentally-extended economic model and a biophysical model and found that food demand in the US accounted for 28% of freshwater withdrawals, 25% of total land area, 18% of greenhouse gas emissions (GHGs), but only 8.6% of gross domestic product (GDP). More recently, He et al. (11) showed that that shifts toward healthier diets can reduce some, but not all, environmental impacts but may be unaffordable for some lower-income groups.

As consumers continue to seek ways to improve the sustainability of their diets, these analyses will continue to rise in importance. NHANES is the backbone of diet sustainability analyses in the US because it is the richest source of nationally representative dietary data. Survey respondents typically report consumption of mixed dishes that contain multiple ingredients, so food composition databases are used to quantify these ingredients, which provides a crosswalk to environmental and economic databases (9). Key among these food composition databases is the Food Commodity Intake Database (FCID), which disaggregates NHANES foods into nearly 500 highly differentiated ingredients and has been used to evaluate dietary intake (12–16), chemical exposure (17–20), environmental impacts (7, 21–25), agricultural resource use (26, 27), and food expenditures (28, 29). However, FCID has not been updated since 2010, so it does not link with more contemporary NHANES data and therefore presents a barrier for further diet sustainability analyses.

To address this research need, the objectives of this study are to (1) link FCID 2001–2010 to NHANES 2011–2018, (2) compare daily per capita food intake with and without these novel linkages, and (3) make these linkages publicly available for use by other researchers.

## Methods

### National Health and Nutrition Examination Survey (NHANES)

Data on individual-level food intake were acquired from NHANES, 2001–2018. NHANES is a continuous, multistage, cross-sectional survey of individual-level food intake, health behaviors, health status, and sociodemographics. Data are collected from ~5,000 non-institutionalized individuals per year using in-person surveys, physical examinations, and laboratory tests performed by trained staff. Data have been collected continuously since 1999 and are released in 2-year cycles (30). Respondents are assigned survey weights that reduce the potential for bias from differential probabilities of selection and nonresponse, and some demographic groups are oversampled to increase the reliability and precision of subgroup analyses (31). The dietary component of NHANES is What We Eat In America, which captures intake of ~4,500 different foods. A portion of these foods are updated for each NHANES survey cycle to reflect new products that enter the market and reformulations of existing products.

### Food Commodity Intake Database

Data on the ingredient composition of NHANES mixed dishes were acquired from Food Commodity Intake Database (FCID), 2001–2010. FCID was developed by the US Environmental Protection Agency (US EPA) to estimate dietary exposure to pesticides when used in conjunction with the Dietary Exposure Evaluation Model (DEEM), and to estimate food consumption rates provided in EPA's Exposure Factors Handbook. FCID provides the gram weight of nearly 500 ingredients present in each NHANES mixed dish in their as consumed forms, which were determined by EPA staff using popular, regional, and specialty cookbooks, as well as professional judgement.

### Matching Procedure

For each new NHANES cycle, many of the foods are retained from previous cycles but some are replaced with new foods to account for changes in the food supply. Therefore, NHANES 2011–2018 includes many foods that are not included in FCID 2001–2010 (Supplementary Table 1). To identify these unmatched foods, NHANES 2011–2018 was merged with FCID 2001–2010 and the unmatched foods were flagged (*n* = 1,656 foods in 2011–2012, *n* = 1,197 in 2013–2014, *n* = 978 in 2015–2016, and *n* = 209 in 2017–2018; total *n* = 4,040). For each unmatched food in NHANES 2011–2018, two investigators independently matched it with a unique food in FCID 2001–2010 based on professional judgement. Perfect agreement between the investigators was achieved for 60% of the foods, and the remaining discrepancies were minor (e.g., the NHANES food was “pizza, with cheese and extra vegetables, not specified as to type of crust,” yet investigator 1 matched it with “pizza with cheese and extra vegetables, regular crust” and investigator 2 matched it with “pizza, cheese, with vegetables, not specified as to type of crust”). All matches were audited by a third investigator who resolved discrepancies (40% of matches) and flagged instances in which investigators 1 and 2 agreed but a closer match was available (< 1% of matches). A fourth investigator reviewed all matches for accuracy. After the discrepancies were resolved, 100% of the NHANES foods were linked with FCID ingredient composition data.

### Statistical Analyses

All FCID ingredients (*n* = 484) were grouped into 21 food categories (Supplementary Table 2) for analysis based on the Healthy Dietary Patterns in the Dietary Guidelines for Americans (32), and more specific categories were established where possible (for example, meat was further categorized into beef, pork, and other meat). Mean per capita intake of each food category was estimated for each NHANES cycle from 2001 to 2018. Temporal trends from 2011 to 2018 were estimated with and without FCID updates using linear regression models, and were compared using paired Wald tests with *P* < 0.05. Respondents with incomplete dietary data were identified by trained NHANES staff and were excluded from the analyses. To ensure equal sample sizes for analytic comparisons between updated and non-updated intakes for each food category, additional respondents were deemed to have incomplete data if they did not consume any foods included in NHANES 2001–2010. All analyses were adjusted for age (continuous), gender (male/female), and energy intake (continuous) using linear regression. Stata 16.1 (Stata Corp; College Station, TX) was used for data management and statistical analyses.

### Data Availability

The updated FCID database is available for download at Data Archiving and Networking Services (DANS) through a Creative Commons license (CCO-1.0). doi: 10.17026/dans-zqx-a23v.

## Results

A total of 80,596 respondents provided dietary data from 2001 to 2018. Individuals < 20 y (*n* = 36,097) and with incomplete dietary data (*n* = 5) were excluded, resulting in a final sample of 44,494 respondents. The majority of respondents were 31–70 y (68%), non-Hispanic white (68%), had income-to-poverty ratios ≥ 2.00 (65%), and completed at least some college (60%; Table 1). Approximately half (52%) were female.

**Table 1:** Characteristics of study sample, 2001–2018 (*n* = 44,494).

**Characteristic**	**n^a^**	**Percent**	
		**(95% CI)^b^**	
Age (y)	44,494		
20–30		21.0	(20.2–22.0)
31–50		37.0	(36.0–38.0)
51–70		30.8	(29.9–31.7)
71+		11.1	(10.6–11.7)
Gender	44,494		
Men		48.1	(47.6–48.6)
Women		51.9	(51.4–52.4)
Race-ethnicity	44,494		
Non-hispanic white		68.2	(65.9–70.3)
Non-hispanic black		11.3	(10.2–12.6)
Hispanic		8.3	(7.3–9.6)
Other		12.2	(11.2–13.2)
Education	44,447		
Less than high school		16.4	(15.5–17.3)
High school or equivalent		24.0	(23.1–24.9)
Some college		31.5	(30.7–32.3)
College graduate		28.1	(26.7–29.6)
Income-to-poverty ratio	40,962		
<0.75		9.0	(8.3–9.7)
0.75–1.30		12.9	(12.2–13.7)
1.31–1.99		13.3	(12.7–13.9)
2.00–3.99		28.7	(27.7–29.6)
4.00+		36.2	(34.7–37.7)

a *Sample sizes are unweighted*.

b *Percentages within each column are adjusted for survey weight*.

**Figure 1:**
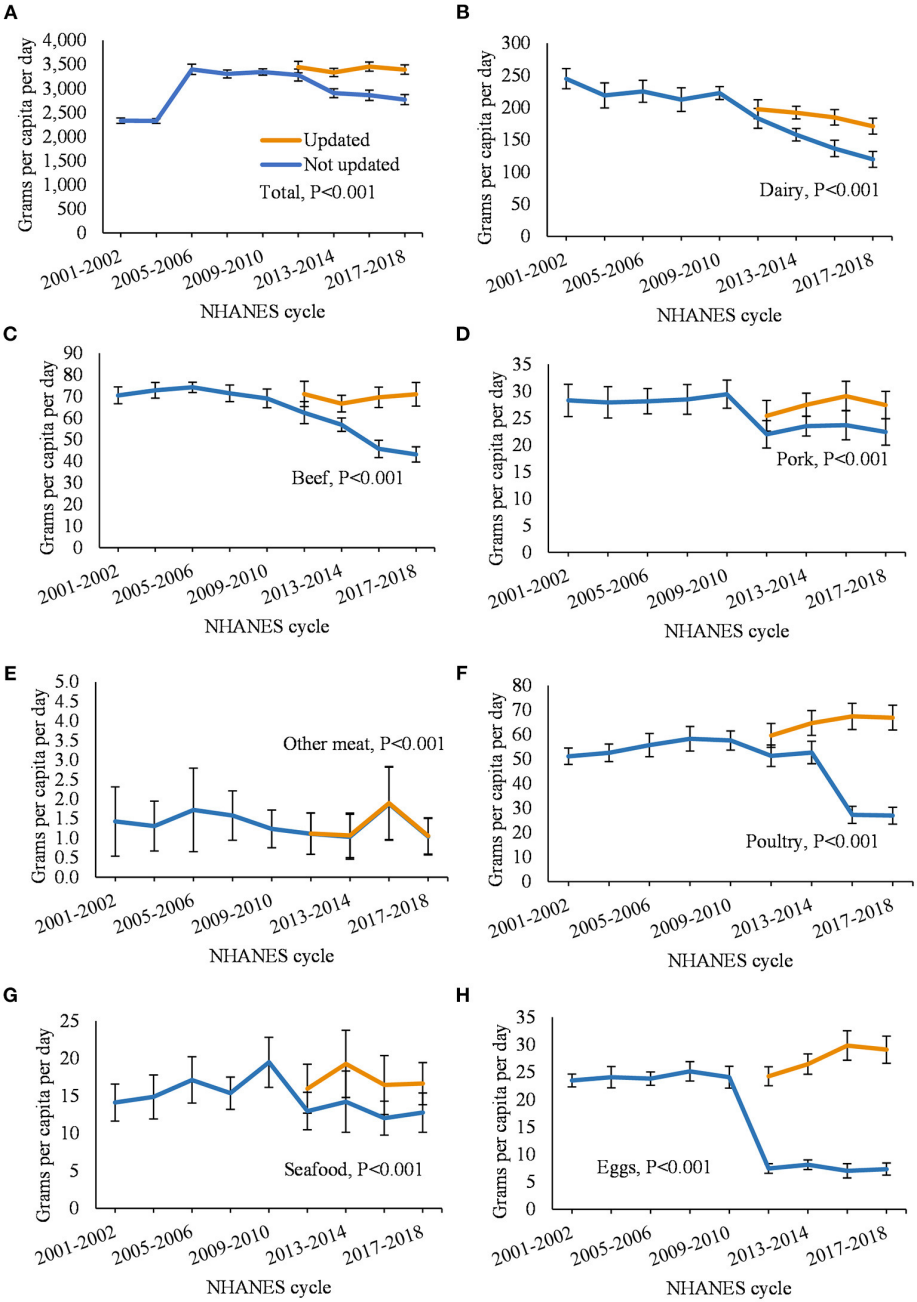
Annual per capita food intake among US adults from 2001 to 2018 comparing updated and non-updated Food Commodity Intake Database (FCID). **(A)** total; **(B)** dairy; **(C)** beef; **(D)** pork; **(E)** other meat; **(F)** poultry; **(G)** seafood; and **(H)** eggs. FCID, Food Commodity Intake Database. Differences between trend lines were evaluated using paired Wald tests adjusted for age, gender, energy intake. Data from 2009 to 2010 were used as the regression intercept.

**Figure 2:**
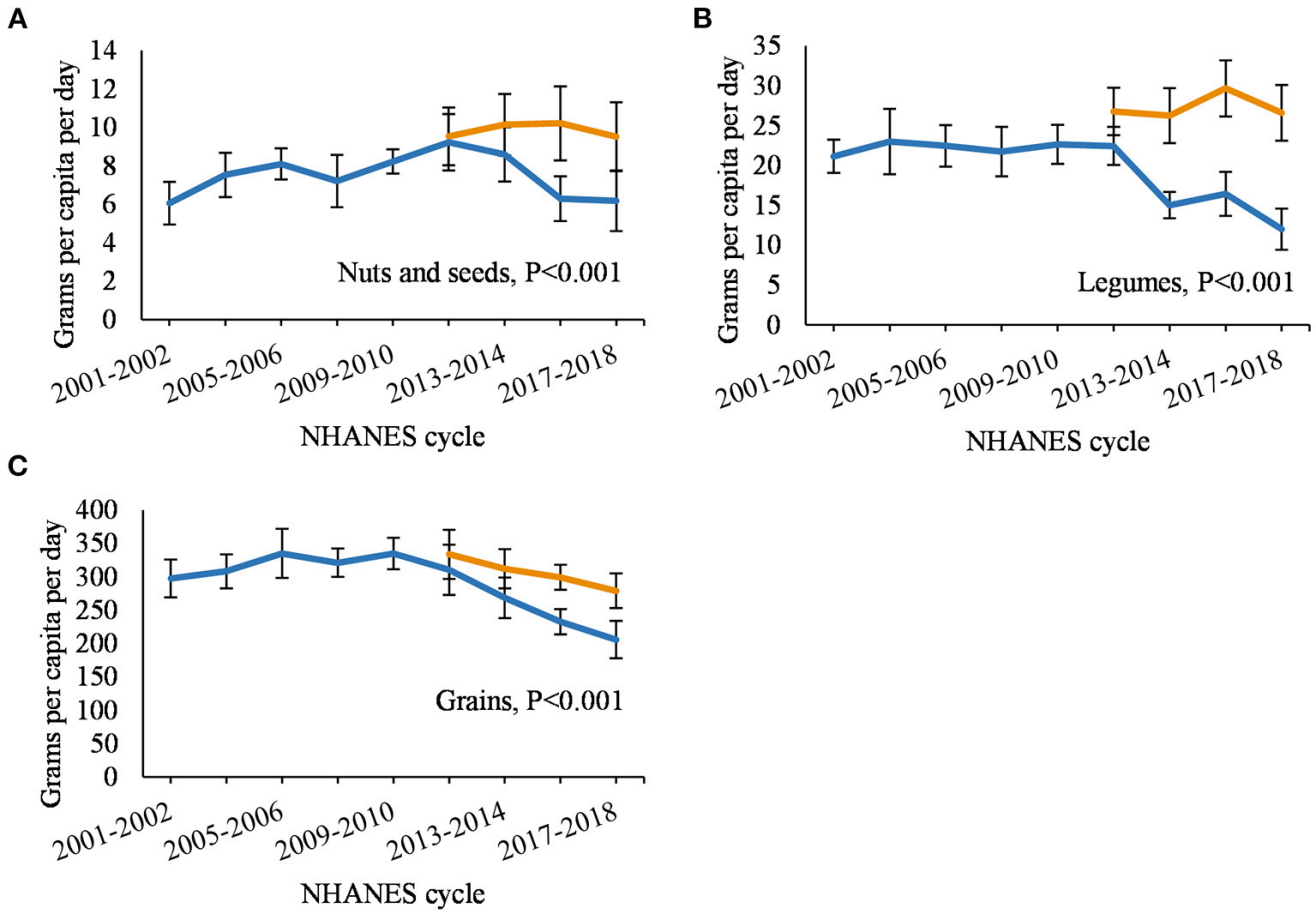
Annual per capita food intake among US adults from 2001 to 2018 comparing updated and non-updated Food Commodity Intake Database (FCID). **(A)** nuts and seeds; **(B)** legumes; and **(C)** grains. FCID, Food Commodity Intake Database. Differences between trend lines were evaluated using paired Wald tests adjusted for age, gender, energy intake. Data from 2009 to 2010 were used as the regression intercept.

Figure 1 displays the annual per capita intake of each food category using updated and non-updated FCID, and Supplementary Table 2 additionally displays the percent difference between the updated and non-updated estimates. Intake of all foods (Figure 1A) from 2011 to 2018 was 5–23% higher per year using the updated FCID compared to the non-updated version (*P* < 0.001 for comparison of temporal trends). Among animal-sourced foods (Figures 1B–H), the updated estimates for dairy were 8–43% higher per year; beef, 14–65%; pork, 16–23%; other meat, 1–3%; poultry, 16–148%; seafood, 233–7%; and eggs, 225–324% (*P* < 0.001 for all temporal comparisons). Among plant-based protein foods (Figures 2A,B), the updated estimates for nuts and seeds were 3–62% higher per year and the updated estimates for legumes were 19–121% higher (*P* < 0.001 for all temporal comparisons). The updated estimates for grains (Figure 2C) were 8–36% higher per year than the non-updated estimates (*P* > 0.001 for temporal comparison).

**Figure 3:**
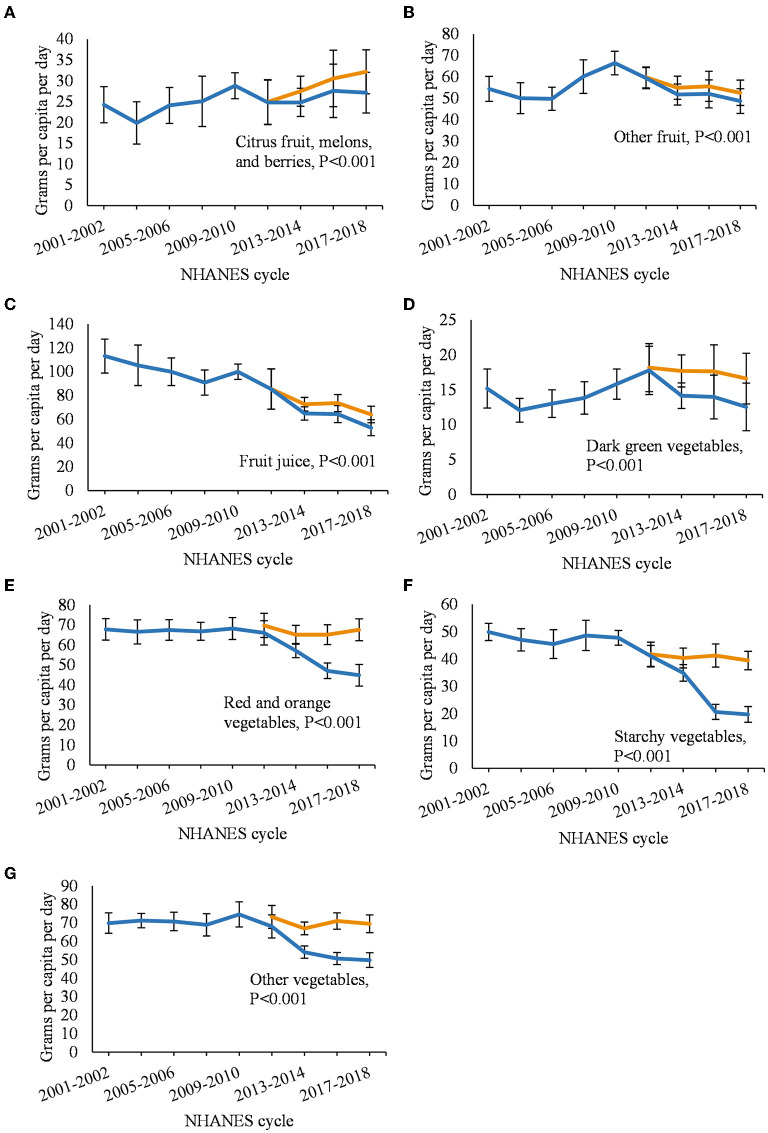
Annual per capita food intake among US adults from 2001–2018 comparing updated and non-updated Food Commodity Intake Database (FCID). **(A)** citrus fruit, melons, and berries; **(B)** other fruit; **(C)** fruit juice; **(D)** dark green vegetables; **(E)** red and orange vegetables; **(F)** starchy vegetables; and **(G)** other vegetables. FCID, Food Commodity Intake Database. Differences between trend lines were evaluated using paired Wald tests adjusted for age, gender, energy intake. Data from 2009 to 2010 were used as the regression intercept.

**Figure 4:**
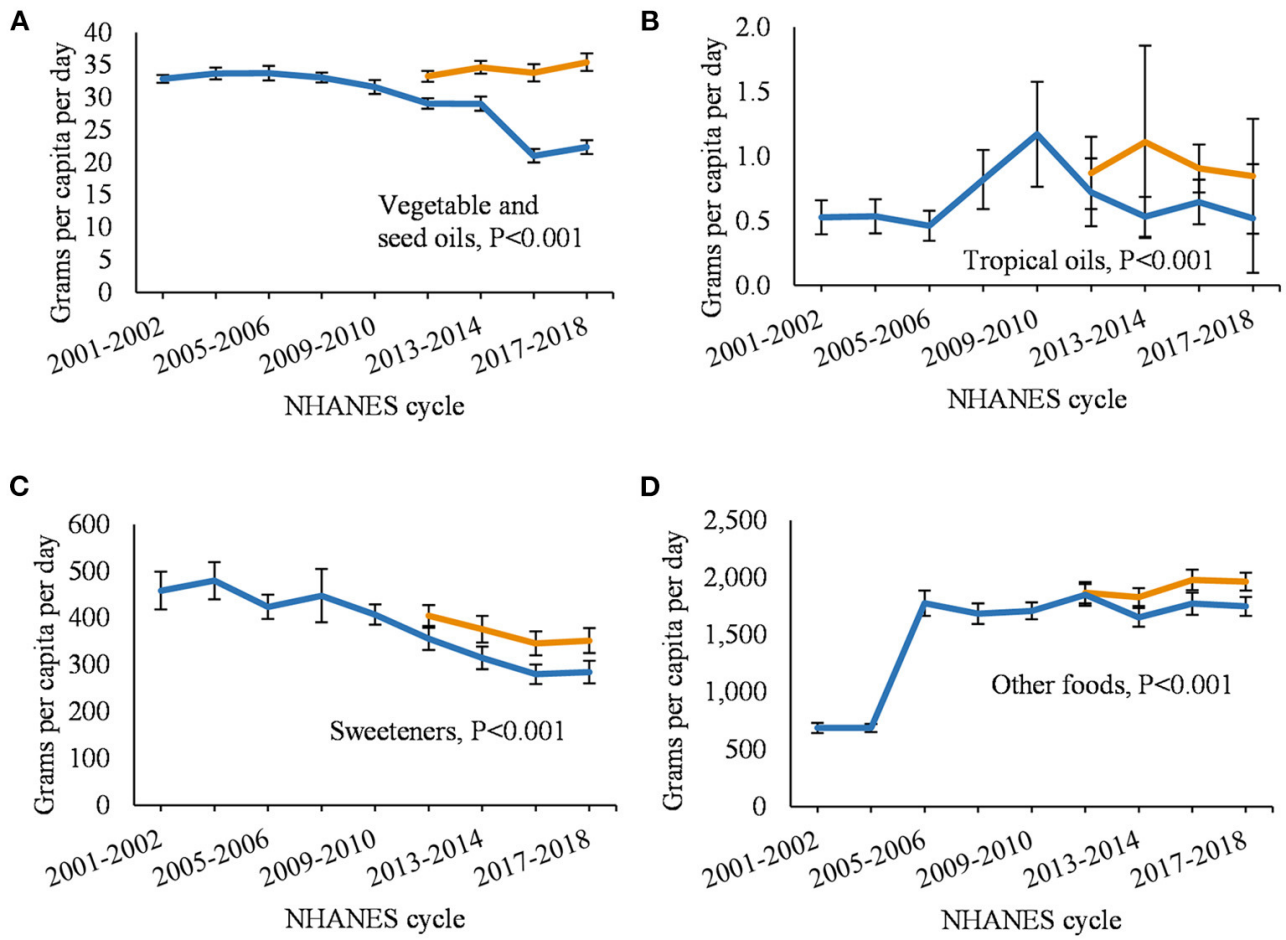
Annual per capita food intake among US adults from 2001 to 2018 comparing updated and non-updated Food Commodity Intake Database (FCID). **(A)** vegetable and seed oils; **(B)** tropical oils; **(C)** sweeteners; and **(D)** other foods. FCID, Food Commodity Intake Database. Differences between trend lines were evaluated using paired Wald tests adjusted for age, gender, energy intake. Data from 2009 to 2010 were used as the regression intercept.

Among fruits (Figures 3A–C), the updated estimates for citrus, melons, and berries were 0–18% higher per year; other fruit, 1–8%; and fruit juice, 0–21% (*P* < 0.001 for all temporal comparisons). Among vegetables (Figures 3D–G), the updated estimates for dark green vegetables were 2–32% higher per year; red and orange vegetables, 6–51%; starchy vegetables, 2–100%; and other vegetables, 8–40% (P < 0.001 for all temporal comparisons). Among oils (Figures 4A,B), the updated estimates were 14–61% higher for vegetable and seed oils, and 21–109% higher for tropical oils. The updated estimates were 14–24% higher for sweeteners (Figure 4C) and 1–12% higher for other foods (Figure 4D; *P* < 0.001 for all temporal comparisons).

## Discussion

For the first time, this study integrated data on food composition from the Food Commodity Intake Database (FCID) with data on food intake from the National Health and Nutrition Examination Survey (NHANES) 2011–2018. Using dietary data from nearly 45,000 individuals, this study demonstrated that total food intake estimated with FCID would be 5–23% lower without these updates, and larger differences were observed for certain food categories. These data are made publicly available for use by other researchers to catalyze advancements in diet sustainability science.

Other food composition databases are available to disaggregate NHANES mixed dishes into their component ingredients, but these have limitations that are now overcome with FCID (Supplementary Table 4). Food Intakes Converted to Retail Commodities Database (FICRCD) has not been updated since 2008 (33) and may require imputation to fill in missing food recipes (34), although the embedded computations on food processing conversions may still be useful for specific research purposes (35). Others (11) have used the Food and Nutrient Database for Dietary Studies (FNDDS) (36) and Food Patterns Equivalents Database (FPED) (37) to disaggregate NHANES foods for diet sustainability analyses, but these databases do not account for food waste which represents ~30% (by weight) of food available for consumption (26), and will underestimate the associated sustainability outcomes. By contrast, FCID is the only food composition database that disaggregates NHANES mixed dishes into ingredients that map onto agricultural commodities, which can then be linked with data on food waste from the Loss-adjusted Food Availability data series (38), as described elsewhere (9). These linked FCID-LAFA data can be used to evaluate the association between food waste and multiple indicators of sustainability, including agricultural resource use (26, 27), environmental impacts (25), diet quality (26, 27), and consumer food expenditures (28, 29). The present study allows these linkages to be extended to more contemporary data on food intake from NHANES 2011–2018, thereby filling an important data gap.

When using the non-updated FCID to estimate food intake, consumption of nearly all food categories decreased from 2011 to 2018 due to incomplete linkages with NHANES. The proportion of NHANES foods not matched with FCID ingredients increased with each NHANES cycle and reached 57% by 2017–2018, which resulted in lower intakes over time for many food categories. The updated database filled those linkage gaps and increased estimates by up to 65% for 16 out of 21 food categories and over 100% for the remaining 5 food categories. The largest changes were observed for eggs (up to 324% increase) and poultry (up to 148% increase), possibly due to their increased use as an ingredient in processed foods that had entered the market since FCID was last updated in 2010 (see below). Temporal trends using the updated database were consistent with estimates of loss-adjusted per capita food availability for all food categories, although a minor discrepancy was observed for other vegetables (38). Other vegetables is a heterogeneous category and LAFA only includes a subset of those included in FCID.

Approximately 20,000 new food products entered the US marketplace every year from 2011 to 2018 (39), and a portion of these were included in each new NHANES cycle. This study linked these foods with proxy recipes that were already included in FCID 2001–2010 rather than create new recipes, and it is possible that new recipes would have increased estimates of food intake even further than what was observed in the present study. Researchers have several options for addressing this limitation. First, new recipes can be created for processed foods that entered the US food supply since 2011, just as EPA did when FCID was updated in 2005 and 2010 (this explains why intake of foods in the “other” category increased dramatically in 2005, which led to an increase in total food intake at that time). Second, researchers can derive the intake of some food categories in mass quantity from other food composition databases, like FICRCD, FNDDS, and FPED (described above).

FCID can be used to estimate the environmental impacts of dietary patterns by linking with the database of Food Impacts on the Environment for Linking to Diets (data FIELD), which provides data on GHG emissions and energy use associated with the production of each FCID ingredient (21). DataFIELD was created by aggregating impact data from a review of life cycle assessments (LCA) that evaluated impacts from cradle-to-farm gate for most ingredients and cradle-to-processing for others, and therefore these data do not include the impacts that occur downstream in the food system (e.g., manufacturing and home cooking). A similar approach has been adopted by others (23). These system boundaries were adopted due to the use of FCID as a crosswalk between LCAs and NHANES, as well as limited data availability from LCA studies on downstream impacts (21). Future efforts will be needed to update food impact estimates with new system boundaries as the LCA literature continues to expand. Limited data linkage between FCID 2001–2010 and NHANES 2011–2018 may have impacted prior sustainability analyses. In some cases, researchers only used NHANES data up until 2010 to align with the year FCID was last updated (21, 25, 40), which does not reflect changes in food consumption that have occurred since that time. Others have combined FCID 2001–2010 with NHANES data up to 2016 with (28, 29) and without (26) imputation to fill data gaps, and demonstrated that incomplete linkages led to a reduction of daily Total Food Demand (sum of retail loss and consumer purchase amount) by 10% (26) and a reduction of daily consumer food expenditures by 7 (28) to 15% (29).

This study has several strengths. To reduce bias in the data linkage procedure, matches were completed independently by two investigators, discrepancies were reconciled by a third investigator, and data were audited by a fourth investigator. Ingredients were categorized into 21 distinct food categories to further investigate bias within each food category, and the raw data are made publicly available so that others can create their own food categories to address specific research questions. Data on food intake were acquired from a large, nationally representative sample over an 18-year time period, which increases generalizability. Finally, this study fills an important research gap by providing ingredient recipes for contemporary NHANES data, which removes a barrier to further diet sustainability analyses.

This study also has limitations. The data linkage procedure was performed by hand coding over 4,000 NHANES foods to nearly 500 FCID ingredients, so misclassification bias cannot be ruled out. This hand-coding method was tested against automated natural language processing during the design phase of this study, and the hand coding method demonstrated superior performance when audited by investigators. Nonetheless, it is possible that further refinement of automated methods may yield similar or improved outcomes; further investigation is warranted to reduce bias and investigator burden. This study used proxy recipes that were already included in FCID 2001–2010 rather than create new recipes for NHANES foods, which may not reflect new products and reformulations that entered the US food supply since 2011. Further efforts are needed by the federal government or others to create new recipes for newly added NHANES foods, which may increase estimates of food intake beyond what was demonstrated in this study. Therefore, the results presented in this study should be considered conservative. Finally, self-reported dietary data are subject to social desirability bias and may have introduced measurement error.

## Conclusions

This study removes a barrier to future diet sustainability analyses by linking data on food composition from FCID 2001–2010 with nationally representative data on food intake from NHANES 2011–2018. As a result, contemporary dietary data can be linked to publicly available data on agricultural resource use, environmental impacts, consumer food expenditures, and other sustainability indicators, which was not previously possible. All data are made publicly available.

## Data Availability Statement

The datasets presented in this study can be found in online repositories. The names of the repository/repositories and accession number(s) can be found below: Data Archiving and Networking Services (DANS) through a Creative Commons license (CCO-1.0). doi: 10.17026/dans-zqx-a23v.

## Ethics Statement

The data collection protocol for the National Health and Nutrition Examination Survey was reviewed and approved by the National Center for Health Statistics Review Board. The patients/participants provided their written informed consent to participate. The data analysis protocol for the present study was reviewed and approved by the Institutional Review Board at William & Mary.

## Author Contributions

ZC was responsible for data management and analysis, designed the research, and wrote the paper. ZC, AC, CK, EJ, BH, JL, AM, MS, and TW conducted the research. All authors read, edited, and approved the final manuscript.

## Funding

This work was supported by the Commonwealth Center for Energy and the Environment at William & Mary and the Interdisciplinary Research Innovation Fund (RAFINS) at the Friedman School of Nutrition Science and Policy at Tufts University.

## Conflict of Interest

ZC received grant support from the Institute for the Advancement of Food and Nutrition Sciences for projects unrelated to the present research, and received honoraria from MKYoung Food & Nutrition Strategies, National Geographic Society, and Nutrition Today for professional activities. The remaining authors declare that the research was conducted in the absence of any commercial or financial relationships that could be construed as a potential conflict of interest.

## Publisher's Note

All claims expressed in this article are solely those of the authors and do not necessarily represent those of their affiliated organizations, or those of the publisher, the editors and the reviewers. Any product that may be evaluated in this article, or claim that may be made by its manufacturer, is not guaranteed or endorsed by the publisher.

## References

[1] Reinhardt SL Boehm R Blackstone NT El-Abbadi NH McNally Brandow JS Taylor SF. Systematic review of dietary patterns and sustainability in the United States. Adv Nutr. (2020) 11:1016–31. 10.1093/advances/nmaa026 32167128 PMC7360461

[2] United Nations High High Level Panel of Experts on Food Security and Nutrition. Nutrition and Food Systems: A Report by the High Level Panel of Experts on Food Security and Nutrition. Rome: United Nations, High Level Panel of Experts on Food Security and Nutrition (2017). p. 152.

[3] United Nations. Sustainable Healthy Diets: Guiding Principles. Rome: Food and Agriculture Organization and World Health Organization (2019).

[4] Rose D Heller MC Roberto CA. Position of the society for nutrition education and behavior: the importance of including environmental sustainability in dietary guidance. J Nutr Educ Behav. (2019) 51:3–15.e1. 10.1016/j.jneb.2018.07.006 30635107 PMC6326035

[5] Mazac R Renwick K Seed B Black JL. An approach for integrating and analyzing sustainability in food-based dietary guidelines. Front Sustain Food Syst. (2021) 5:544072. 10.3389/fsufs.2021.544072

[6] International Food Information Council. Food and Health Survey. International Food Information Council (2020).

[7] Willits-Smith A Aranda R Heller MC Rose D. Addressing the carbon footprint, healthfulness, and costs of self-selected diets in the USA: a population-based cross-sectional study. Lancet Planet Health. (2020) 4:e98–106. 10.1016/S2542-5196(20)30055-3 32220679 PMC7232940

[8] Willett W Rockstrom J Loken B Springmann M Lang T Vermeulen S. Food in the Anthropocene: the EAT-Lancet Commission on healthy diets from sustainable food systems. Lancet. (2019) 393:447–92. 10.1016/S0140-6736(18)31788-4 30660336

[9] Conrad Z Stern A Love DC Salesses M Cyril A McDowell A. Data integration for diet sustainability analyses. Sustainability. (2021) 13:8082. 10.3390/su1314808230795483

[10] Canning P Rehkamp S Hitaj C Peters C. Resource Requirements of Food Demand in the United States. US Department of Agriculture. Economic Research Service (2020).

[11] He P Feng K Baiocchi G Sun L Hubacek K. Shifts towards healthy diets in the US can reduce environmental impacts but would be unaffordable for poorer minorities. Nat Food. (2021) 2:664–72. 10.1038/s43016-021-00350-5 37117464

[12] Raatz SK Conrad Z Jahns L Belury MA Picklo MJ. Modeled replacement of traditional soybean and canola oil with high-oleic varieties increases monounsaturated fatty acid and reduces both saturated fatty acid and polyunsaturated fatty acid intake in the US adult population. Am J Clin Nutr. (2018) 108:594–602. 10.1093/ajcn/nqy127 30084912

[13] Casperson S Conrad Z Raatz S Jahns L Roemmich JN Picklo MJ. Impact of beef consumption on saturated fat intake in the United States adult population: insights from modeling the influence of bovine genetics and nutrition. Meat Sci. (2020) 169:108225 10.1016/j.meatsci.2020.108225 32629167

[14] Kim H Rebholz CM Caulfield LE Ramsing R Nachman KE. Trends in types of protein in US adults: results from the national health and nutrition examination survey 1999-2010. Public Health Nutr. (2019) 22:191–201. 10.1017/S1368980018003348 30587270 PMC6597323

[15] Ba DM Gao X Al-Shaar L Muscat J Chinchilli VM Ssentongo P. Prospective study of dietary mushroom intake and risk of mortality: results from continuous national health and nutrition examination survey (NHANES) 2003-2014 and a meta-analysis. Nutr J. (2021) 20:80. 10.1186/s12937-021-00738-w 34548082 PMC8454070

[16] Kim H Caulfield LE Rebholz CM Ramsing R Nachman KE. Trends in types of protein in US adolescents and children: Results from the National Health and Nutrition Examination Survey 1999–2010. PLoS ONE. (2020) 15:e0230686. 10.1371/journal.pone.0230686 32214368 PMC7098572

[17] Hyland C Kogut K Gunier RB Castorina R Curl C Eskenazi B. Organophosphate pesticide dose estimation from spot and 24-hr urine samples collected from children in an agricultural community. Environ Int. (2021) 146:106226 10.1016/j.envint.2020.106226 33152651 PMC8168949

[18] Nachman KE Love DC Baron PA Nigra AE Murko M Raber G. Nitarsone, inorganic arsenic, and other arsenic species in Turkey meat: Exposure and risk assessment based on a 2014 U.S. market basket sample. Environ Health Perspect. (2017) 125:363–9. 10.1289/EHP225 27735789 PMC5332177

[19] Awata H Linder S Mitchell LE Delclos GL. Association of dietary intake and biomarker levels of arsenic, cadmium, lead, and mercury among Asian populations in the United States: NHANES 2011–2012. Environ Health Perspect. (2017) 125:314–23. 10.1289/EHP28 27586241 PMC5332183

[20] Nigra AE Nachman KE Love DC Grau-Perez M Navas-Acien A. Poultry consumption and arsenic exposure in the U S population. Environ Health Perspect. (2017) 125:370–7. 10.1289/EHP351 27735790 PMC5332189

[21] Heller MC Willits-Smith A Meyer R Keoleian GA Rose D. Greenhouse gas emissions and energy use associated with production of individual self-selected US diets. Environ Res Lett. (2018) 13:044004. 10.1088/1748-9326/aab0ac 29853988 PMC5964346

[22] Heller MC Willits-Smith A Mahon T Keoleian GA Rose D. Individual US diets show wide variation in water scarcity footprints. Nat Food. (2021) 2:255–63. 10.1038/s43016-021-00256-2 37118462

[23] Bozeman JF III Ashton WS Theis TL. Distinguishing environmental impacts of household food-spending patterns among U.S. Demographic Groups. Environ Engineer Sci. (2019) 36:763–77. 10.1089/ees.2018.0433

[24] Bozeman JF Bozeman R Theis TL. Overcoming climate change adaptation barriers: a study on food–energy–water impacts of the average American diet by demographic group. J Ind Ecol. (2020) 24:383–99. 10.1111/jiec.12859

[25] Rose D Heller MC Willits-Smith AM Meyer RJ. Carbon footprint of self-selected US diets: nutritional, demographic, and behavioral correlates. Am J Clin Nutr. (2019) 109:526–34. 10.1093/ajcn/nqy327 30698631 PMC6408204

[26] Conrad Z Blackstone NT Roy ED. Healthy diets can create environmental trade-offs, depending on how diet quality is measured. Nutr J. (2020) 19:117. 10.1186/s12937-020-00629-6 33109207 PMC7592508

[27] Conrad Z Niles MT Neher DA Roy ED Tichenor NE Jahns L. Relationship between food waste, diet quality, and environmental sustainability. PLoS ONE. (2018) 13:e0195405. 10.1371/journal.pone.0195405 29668732 PMC5905889

[28] Conrad Z. Daily cost of consumer food wasted, inedible, and consumed in the United States, 2001–2016. Nutr J. (2020) 19:35. 10.1186/s12937-020-00552-w 32306976 PMC7168972

[29] Conrad Z Reinhardt S Boehm R McDowell A. Higher diet quality is associated with higher diet costs when eating at home and away from home: national health and nutrition examination survey, 2005–2016. Public Health Nutr. (2021) 24:5047–57. 10.1017/S1368980021002810 34176554 PMC11082814

[30] US US Department of Health and Human Services Centers for Disease Control and Prevention (CDC). About the National Health and Nutrition Examination Survey. CDC (2017).

[31] Chen T Clark J Riddles M Mohadjer L Fakhouri T. The National Health and Nutrition Examination Survey, 2015–2018: sample design and estimation procedures. National Center for Health Statistics. Vital Health Stat. (2020) 2:1–35. 33663649

[32] US Department of Health and Human Services and US Department of Agriculture. Dietary Guidelines for Americans 2020–2025, Appendix 3. Washington, DC: US Department of Health and Human Services and US Department of Agriculture (2020).

[33] US US Department of Agriculture Agricultural Research Service. Food Intakes Converted to Retail Commodities Database. US Department of Agriculture, Agricultural Research Service (2021).

[34] Lin N-H. COVID-19 Working Paper: Shares of Commodity Consumption at Home, Restaurants, Fast Food Places, Schools, and Other Away-from-Home Places: 2013–16. *US Contract No. AP-085*. Department of Agriculture. Economic Research Service (2020).

[35] Kovacs B Miller L Heller MC Rose D. The carbon footprint of dietary guidelines around the world: a seven country modeling study. Nutr J. (2021) 20:15. 10.1186/s12937-021-00669-6 33648497 PMC7923667

[36] US US Department of Agriculture Agricultural Research Service. Food and Nutrient Database for Dietary Studies. US Department of Agriculture, Agricultural Research Service (2021).

[37] US US Department of Agriculture Agricultural Research Service. Food Patterns Equivalents Database (FPED). US Department of Agriculture, Agricultural Research Service (2021).

[38] US US Department of Agriculture Economic Research Service. Loss-adjusted Food Availability (LAFA) Data Series. US Department of Agriculture, Economic Research Service (2021).

[39] US US Department of Agriculture Economic Research Service. Food Markets and Prices: New Products. US Department of Agriculture, Economic Research Service (2021).

[40] Bozeman JF Springfield S Theis TL. Meeting EAT-Lancet food consumption, nutritional, and environmental health standards: a U.S. case study across racial and ethnic subgroups. Environ Justice. (2020) 13:160–72. 10.1089/env.2020.0018 33101580 PMC7580058

